# Independent testing infrastructure for new approach methodologies: the UC Davis NAMs testing center

**DOI:** 10.3389/fmedt.2026.1966220

**Published:** 2026-09-04

**Authors:** K. C. Kent Lloyd

**Affiliations:** Department of Surgery, School of Medicine and Mouse Biology Program, University of California, Davis, CA, United States

**Keywords:** biomedical research, context of use, new approach methodologies, reproducibility, translational models, validation, verification

## Abstract

As biomedical research moves toward greater use of human-based experimental systems, the practical problem is no longer simply how to develop new models, but how to determine when they are valid and predictable for a particular use. Independent testing infrastructure can help make that determination. The UC Davis NAMs Testing Center is one model for bringing comparative biology, engineering, clinical and computational expertise together to evaluate NAMs for defined biomedical research purposes.

## Introduction

New Approach Methodologies, or NAMs, include organoids, microphysiological systems, engineered tissues, human cell-based assays, computational models, artificial intelligence and other non-animal approaches. Interest in these methods has expanded from toxicology and chemical safety because they can provide direct access to human-specific biology, increase experimental throughput and support mechanistic studies. From that perspective, there is increasing interest in the use for primary biomedical research applications rather than merely regulatory safety testing. However, objective evidence is needed to establish that a NAM is valid for a particular research purpose before it replaces another experimental approach.

Independent laboratories are being established to address this challenge. One example of these is the UC Davis NAMs Testing Center. This Center is an academic collaborative testing and evaluation resource that works with investigators to determine what a NAM can support under defined conditions and where its limits become important. Its emphasis is on disease modeling, drug target discovery, therapeutic evaluation and interpretation of biological mechanisms. The Center brings together expertise needed to test the extent to which a NAM can address a scientific hypothesis, rather than simply characterizing a NAM as a technical platform. This is the part of NAM development that often becomes difficult once a technology leaves the laboratory in which it was created.

## Why the center is needed today

Much of the vocabulary and standards of NAM validation were developed for toxicological and chemical safety testing, where investigators often ask narrowly defined questions such as whether a chemical causes skin sensitization, genotoxicity or organ injury. Reference chemicals, positive and negative controls and performance metrics can often be specified in advance. This work remains essential and has been advanced through the Interagency Coordinating Committee on the Validation of Alternative Methods (ICCVAM), the former National Interagency Center for the Evaluation of Alternative Test Methods (NICEATM) program and the 2018 ICCVAM Strategic Roadmap (1). In June 2026, NICEATM activities moved into the NIH Office of Research Innovation, Validation, and Application (ORIVA) where the Division of NICEATM (D-NICEATM) now supports NAM evaluation and ICCVAM coordination (2).

On the other hand, biomedical discovery research is often more exploratory and less constrained by predefined endpoints. A researcher studying Alzheimer's disease, cancer, fibrosis, immune dysfunction or metabolic disease may not know which cell type, tissue, developmental stage or systemic interaction will prove most important. A human organoid might reproduce one aspect of a disease while missing immune, endocrine, vascular, neural, metabolic or biomechanical influences. A computational model may predict an outcome in one dataset yet fail in another. A microphysiological system may capture a tissue interface without reproducing whole-body pharmacology.

For biomedical research, NAM evaluation begins with the proposed context of use, considering the scientific question, endpoint, experimental setting and outcome the model is expected to inform. In this article, verification refers to technical evidence that a method performs as specified. Validation refers more broadly to evidence that the method can support a defined inference in its stated context of use. Qualification is used for formal acceptance of a tool for a specified use by a regulatory or other decision-making authority when such a pathway applies. Fit-for-purpose assessment ties the amount and type of evidence to the intended use rather than to a universal validation label. These distinctions are consistent with recent confidence frameworks that emphasize context of use, biological relevance, technical characterization, transparency and independent review (3, 4).

## A complementary role in the validation ecosystem

The Center occupies a different place in this landscape from organizations that set regulatory requirements or confer formal acceptance. ICCVAM and D-NICEATM/ORIVA coordinate federal activities and validation support, FDA's ISTAND program provides a qualification pathway for novel drug development tools used in a defined context of use, and OECD guidance and EURL ECVAM provide international frameworks and validation infrastructure for test methods (3–6). The NIH Complement-ARIE Validation and Qualification Network (VQN) is building a public-private network and standardized evidence packages for NAMs, but it does not itself confer regulatory qualification (7, 8). In this context, the UC Davis Center is intended to function as a hands-on testing node for biomedical research, helping to define a study, carry out independent comparative or transferability testing, and assemble evidence that investigators, funders, industry or, when relevant, established regulatory programs can use. The Center does not confer regulatory acceptance.

## An institutional model for independent testing

Independent NAM testing can require expertise that rarely resides in a single laboratory. The Center is a campus-wide UC Davis resource drawing on extant biomedical engineering, comparative biology, pathology, genetics, imaging, phenotyping, veterinary medicine, human health research and disease modeling. UC Davis assembles these capabilities from an academic health system, the Schools of Medicine and Veterinary Medicine, the Mouse Biology Program, the National Biomedical Research Institute, the Comprehensive Cancer Center and specialized research cores. The Center draws from this diversity of expertise and infrastructure to assemble the pieces needed for a particular question. A steering committee of campus administrators and faculty advisors guides Center priorities, coordinates participation across campus units and advises on testing activities and services (9).

Several capabilities are already operational through participating UC Davis programs and cores. These include patient-derived xenograft (PDX) models, full necropsy and histopathology services, x-ray, microPET and microCT imaging, and *in vivo* phenotyping pipelines spanning cardiovascular, respiratory, neurological and behavioral, ophthalmic, auditory, metabolic and endocrine, digestive, hematological, immune, musculoskeletal, integumentary, renal, reproductive and embryonic/developmental systems. Projects can also draw on transcriptomic, proteomic and metabolomic analyses and, with appropriate approvals, clinical specimens from UC Davis biorepository resources (9–11).

A fit-for-purpose study may need several of these capabilities at once. Depending on the project, evaluation might combine human cells or organoids with quantitative imaging or pathology, molecular profiling, computational analysis and an independent method that measures the relevant biology in a different way (aka orthogonal comparator). The Center's role is to organize that work around the question being tested and to separate developer-generated evidence from independent evaluation. It also provides a point of contact for academic researchers, industry partners and government or nonprofit programs seeking comparative expertise (9).

The Center conducts testing of commercial or proprietary platforms under applicable UC Davis agreements and institutional policies governing confidential information, research data and intellectual property, while preserving the ability of investigators to independently analyze and report the scientific findings. When knowledge of sample identity, experimental condition or expected outcome could influence assessment, samples or data may be coded and evaluated under blinded conditions until predefined analyses are complete. Potential financial or professional conflicts of interest are disclosed and managed through institutional review, recusal or independent oversight as appropriate, so that a developer or commercial sponsor does not determine the interpretation or conclusions of the evaluation.

## What the center does

The Center can engage a project at several stages. Early work often begins by defining the context of use, such as the biological question, endpoint, comparator, performance criteria and limits of interpretation. A technology developer may have a well-developed platform without yet having a precise statement of what the platform can reliably support.

Verification addresses technical reliability. The work can include control performance, repeatability, quality-control criteria, reagent or operator effects and whether the method can be transferred to another laboratory under defined conditions. Interlaboratory transfer or multisite studies are appropriate when the intended use depends on reproducibility beyond the developer's laboratory (4, 12).

Biological evaluation asks whether the model contains the features needed for the domain of inference. A fibrosis model, for example, may require matrix-producing cells, inflammatory signaling and enough time for tissue remodeling. A tumor model intended to predict immune-mediated response must include relevant immune biology, not only cancer-cell growth.

Comparative testing is selected according to the question. A NAM may need comparison with human tissues, clinical data, another NAM, an existing dataset or a carefully designed *in vivo* study. No single comparator serves as a gold standard for every application. The useful comparator is the one that addresses the uncertainty in the intended use.

Discordant results also require interpretation. Disagreement among a NAM, an animal model and human data may point to missing biology, a species difference, an inappropriate exposure condition or a limitation in the comparator. In some cases, this disagreement is the result that most clearly defines the limits of a NAM and where it can and cannot be used.

For example, a developer proposing a human organoid as a disease model might first establish technical performance and transferability, then compare histopathology, imaging or molecular features with the human condition and with an orthogonal system chosen for the specific use. If the model is intended to guide treatment selection, the evidentiary bar would be higher and prospective testing against independent outcomes would be needed. While the testing logic is the common element, the exact combination of assays and comparators changes with the project.

Currently, the Center is supporting studies that integrate patient-derived, 3D-bioprinted tumor models with orthogonal *in vivo* tumor models to evaluate therapeutic response and mechanism. In an ongoing bladder cancer study, responses observed in the 3D platform are compared with an established syngeneic bladder cancer model developed through the Center, while machine-learning analysis of tumor morphology and large human cancer datasets is used to assess mechanism and potential patient relevance. This workflow provides an example of how complementary NAM, animal, and human data can be integrated to evaluate both biological validity and translational relevance.

**Figure 1:**
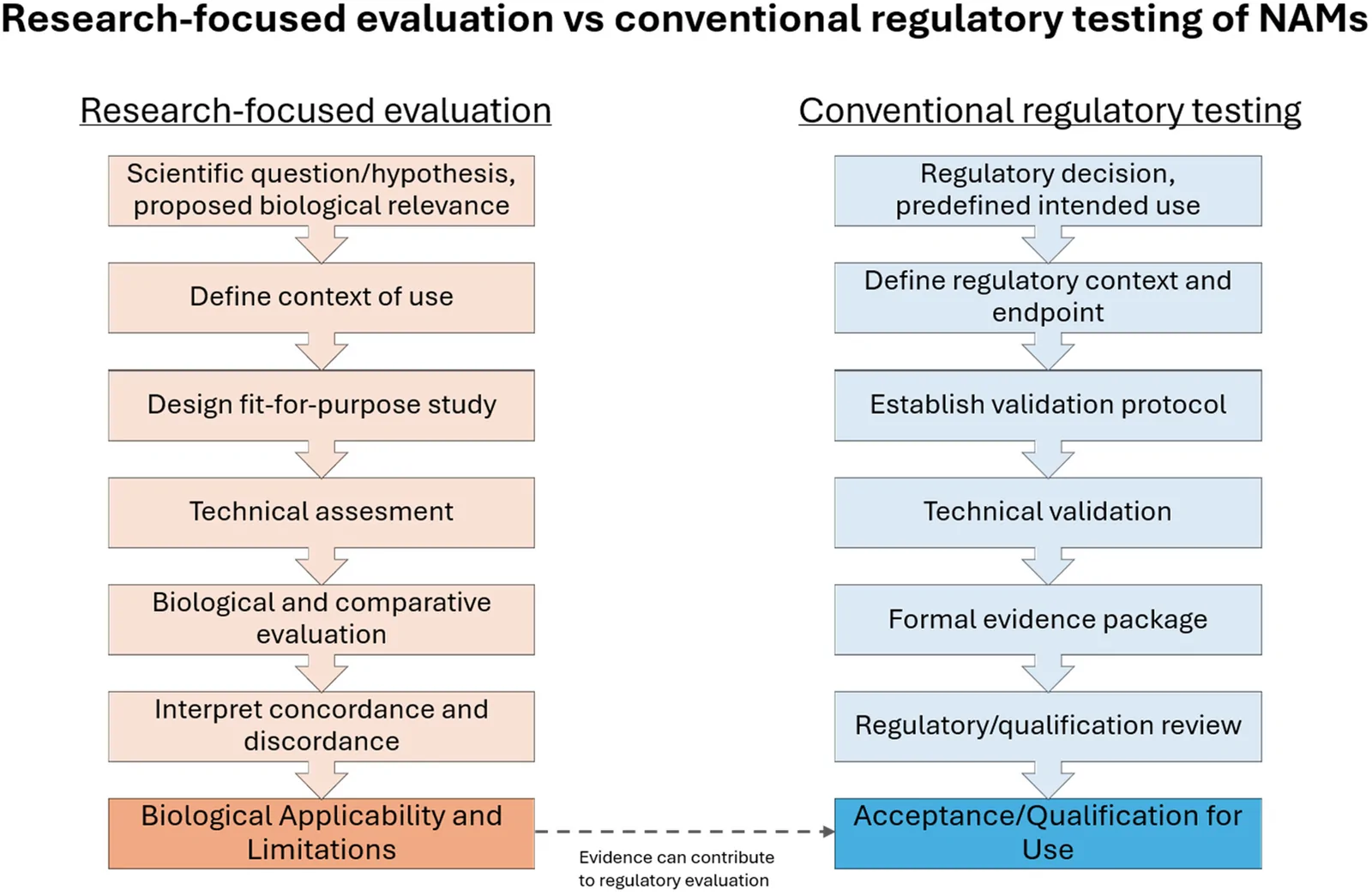
Conceptual comparison of research-focused NAM evaluation at the UC Davis NAMs testing center with a typical regulatory validation or qualification pathway. Research-focused evaluation begins with the scientific question and defines evidence according to the proposed context of use, including technical assessment, biological relevance, comparative testing and interpretation of discordant results. Its principal output is a statement about biological relevance and applicability that describes what the model can and cannot support. Regulatory pathways generally begin with a specified decision context and predefined performance requirements and may culminate in formal acceptance or qualification. Evidence generated through research-focused evaluation can contribute to subsequent regulatory evaluation when appropriate.

The relationship between this research-focused evaluation approach differs from a typical regulatory validation or qualification pathway (Figure 1).

## Why comparative expertise matters

A testing center has to work across model classes because the biological question determines the useful evidence. UC Davis has a long history of developing, preserving and interpreting animal models for biomedical research, and that experience is directly relevant when an investigator needs to know what biology would be lost or gained by moving to a NAM. The objective is to identify the least complex experimental approach that still preserves the scientific value and to recognize when an integrated physiological question requires evidence from an intact organism.

A human neuromuscular junction assay may be able to replace a defined animal potency assay when the relevant mechanism is captured directly. Genome-wide mouse phenotyping programs such as the NIH Knockout Mouse Production and Phenotyping (KOMP2) program (13) and the International Mouse Phenotyping Consortium (14), by contrast, ask organism-level questions in which the relevant tissue or phenotype may not be known in advance. Many projects lie between these examples. NAMs may be used to screen compounds, identify mechanisms or reduce animal numbers, while selected *in vivo* studies address integrated physiology. Comparative expertise helps define which evidence is actually needed for the question (17).

This approach also fits well with current NIH activity. The Complement-ARIE program is supporting development and adoption of human-based NAMs (7) and its VQN is building standardized evidence packages and pilot projects for selected platforms (8). NIH has also launched the Standardized Organoid Modeling Center to address reproducibility and standardization in organoid research (15). ORIVA now coordinates NIH-wide work on development, validation and application of human-based approaches and houses D-NICEATM (2). While these efforts operate at different levels, an independent testing center can contribute project-level experimental evidence within that broader ecosystem.

## How the center can be used

The Center is open to academic investigators, biotechnology and pharmaceutical companies, nonprofit organizations, government scientists, foundations and research consortia. A project can begin with a NAM, a biological question or a specific validation need.

Early consultation is useful because evaluation criteria can be built into development rather than added after a platform is finished. Developers can define the context of use, quality-control criteria, comparators and a prospective testing plan before investing heavily in a system that may later prove difficult to interpret.

More mature technologies can be evaluated independently. A company may seek evidence that an organoid platform predicts a defined therapeutic response, an academic group may want to know whether a disease model reproduces a mechanism observed in patients, or a funder may need to judge whether a NAM is ready for broader adoption. For the Center, useful outcomes include a method that transfers reproducibly, a well-supported domain of inference, or a clear finding that a proposed inference is too broad. A negative or discordant result can therefore be informative if it identifies a limitation before the model is used more widely.

## Discussion: a model for distributed testing

Independent testing centers should make biomedical model evaluation more rigorous, transparent and useful. At UC Davis, the goal is a domain-of-inference statement that describes what a model can support, what remains uncertain and which evidence was used to reach that conclusion. This is more informative for subsequent users than a general label of “validated” for a specific particular technology or model.

Replication and transferability are part of this work. NAMs can make replication more tractable when cell sources, protocols, devices, software and quality-control criteria are standardized and shared, but complex systems also introduce new sources of variability (12). Testing centers can determine whether performance transfers across laboratories and can preserve negative, null and discordant findings that define model limitations. NIH's Replication to Enhance Research Impact Initiative supports independent replication of preclinical, translational and technology-development research, including validation of novel technologies (16).

Independent testing centers should not assume that one model class should replace another by default. Their task is to judge the evidence against the question being asked. In some projects a NAM may replace an animal experiment. In others it may screen candidates or add human-specific mechanistic information while an *in vivo* study addresses integrated physiology. The same questions apply to every model: what can it reliably tell us, and what evidence supports that conclusion?

Trust will determine how quickly useful NAMs are adopted in biomedical research. Independent testing centers can contribute by providing comparative expertise, reproducibility testing and evidence that is separate from the developer's own evaluation. The UC Davis NAMs Testing Center is one example of how those capabilities can be assembled around a defined scientific question. Similar nodes, connected through national and international networks, could provide a practical route from method development to evidence-based use.

## Data Availability

The original contributions presented in the study are included in the article/Supplementary Material, further inquiries can be directed to the corresponding author.
